# Novel Graphical Analyses of Runs of Homozygosity among Species and Livestock Breeds

**DOI:** 10.1155/2016/2152847

**Published:** 2016-10-30

**Authors:** Laura Iacolina, Astrid V. Stronen, Cino Pertoldi, Małgorzata Tokarska, Louise S. Nørgaard, Joaquin Muñoz, Anders Kjærsgaard, Aritz Ruiz-Gonzalez, Stanisław Kamiński, Deirdre C. Purfield

**Affiliations:** ^1^Department of Chemistry and Bioscience, Aalborg University, Section of Biology and Environmental Engineering, Fredrik Bajers Vej 7H, 9220 Aalborg, Denmark; ^2^Aalborg Zoo, Mølleparkvej 63, 9000 Aalborg, Denmark; ^3^Mammal Research Institute Polish Academy of Sciences, Ul. Waszkiewicza 1, 17-230 Białowieża, Poland; ^4^Department of Zoology and Animal Cell Biology, University of the Basque Country UPV/EHU, C/Paseo de la Universidad 7, 01006 Vitoria-Gasteiz, Spain; ^5^Department of Animal Genetics, University of Warmia and Mazury in Olsztyn, 10-718 Olsztyn, Poland; ^6^Animal & Biosciences Department, Animal & Grassland Research and Innovation Centre, Teagasc, Moorepark, Fermoy, County Cork, Ireland

## Abstract

Runs of homozygosity (ROH), uninterrupted stretches of homozygous genotypes resulting from parents transmitting identical haplotypes to their offspring, have emerged as informative genome-wide estimates of autozygosity (inbreeding). We used genomic profiles based on 698 K single nucleotide polymorphisms (SNPs) from nine breeds of domestic cattle (*Bos taurus*) and the European bison (*Bison bonasus*) to investigate how ROH distributions can be compared within and among species. We focused on two length classes: 0.5–15 Mb to investigate ancient events and >15 Mb to address recent events (approximately three generations). For each length class, we chose a few chromosomes with a high number of ROH, calculated the percentage of times a SNP appeared in a ROH, and plotted the results. We selected areas with distinct patterns including regions where (1) all groups revealed an increase or decrease of ROH, (2) bison differed from cattle, (3) one cattle breed or groups of breeds differed (e.g., dairy versus meat cattle). Examination of these regions in the cattle genome showed genes potentially important for natural and human-induced selection, concerning, for example, meat and milk quality, metabolism, growth, and immune function. The comparative methodology presented here permits visual identification of regions of interest for selection, breeding programs, and conservation.

## 1. Introduction

Mating among closely related individuals can affect the fitness of the progeny by increasing the inbreeding coefficient (*F*) [1] and therefore the probability that alleles at a locus, sampled randomly in a population, are identical by descent (IBD) [2]. The reduction in fitness can be due to the accumulation of recessive lethal genetic disorders, reduction of fertility, and lower adaptive potential [1, 3, 4].

In wild living and captive populations, there is an urgent need to reduce inbreeding and augment genetic diversity, and this can be achieved by implementing carefully planned mating strategies. One possibility consists in reducing the level of inbreeding per generation and the response to selection (optimal contribution selection) [5]. The estimation of* F* requires completeness and accuracy of the available pedigree records, which are not always available, because of missing information or registration errors. When genotypes are available a probabilistic approach can be utilized for the reconstruction of the pedigree. However, such an approach does not take into account the stochastic nature of recombination [6]. New approaches based on the runs of homozygosity (ROH), which are DNA segments that harbour uninterrupted stretches of homozygous genotypes, have shown to be reliable estimates of autozygosity at the genome-wide level [7–9].

In addition, the frequency and extent of ROH can be used to estimate the time when the inbreeding event took place. Considering that recombination events break long chromosome segments, it is assumed that long autozygous segments in an individual derive from a common recent ancestor, whereas shorter autozygous segments are indicating a remote common ancestor [10–12]. We should therefore expect that the longer the homozygous segments, the more recent the inbreeding. However, long ROH may also be explained by a recent event under strong selective pressure. ROH can thus be used to identify the genomic signatures of recent and/or ancient selective pressure, as shown by [9]. Additionally, fixed ROH in all the individuals in a population could indicate past selective events. Clearly, the presence of long ROH at relatively high frequency in a population could also indicate the presence of genetic substructure, with consanguineous mating occurring only within some subpopulations [13]. ROH are also affected by demographic events [8] and further investigation should examine issues such as skewed reproductive success.

The objective of this study was to describe and compare the distribution of ROH of different length in nine* Bos taurus* cattle breeds under different management practices and selection histories. The same comparison was made at the interspecific level by comparing the distribution of the ROH between the abovementioned cattle breeds and the Lowland line of the European bison (*Bison bonasus*) from the Białowieża National Park (Poland). The Lowland line is highly inbred due to only seven founders [14].

While previous investigations were exclusively based on the count and sum of the number of ROH above a given length [9], in this paper we analysed the frequency of SNPs falling within a ROH above and below an* a priori* chosen length (15 Mb) and we visualized the different distributions across populations. In addition, this graphical visualization allows the identification of similarities and dissimilarities in the regions that can be used to investigate possible adaptive/selective patterns.

## 2. Material and Methods

### 2.1. Genotypes and Quality Control

Genotypes consisting of 777,972 single nucleotide polymorphisms (SNPs) from the BovineHD BeadChip (Illumina Inc., San Diego, CA) were generated for 891 sires of multiple breeds. Breeds represented include Angus (*n* = 39), Belgian Blue (*n* = 38), Charolais (*n* = 117), Friesian (*n* = 98), Hereford (*n* = 40), Holstein (*n* = 262), Holstein-Friesian crosses (*n* = 111), Limousin (*n* = 128), and Simmental (*n* = 58) (data from [9]). Angus, Belgian Blue, and Hereford are primarily meat breeds; Friesian, Holstein, and Holstein-Friesian crosses are primarily dairy breeds, while Limousin, Simmental, and Charolais are used for both milk and meat. Forty European Lowland bison (*Bison bonasus*) from Białowieża National Park (Poland) were used for comparison. GenomeStudio™ (Illumina Inc., San Diego, CA) and accompanying guidelines from Illumina (http://www.illumina.com/Documents/products/technotes/technote_infinium_genotyping_data_analysis.pdf) were used for quality control. Total individual call rate in the bison was 0.99. For cattle, only biallelic SNPs on the 29 autosomes were retained after removing all monomorphic SNPs across breeds, filtering for Hardy Weinberg Equilibrium (*p* < 0.0001) within each breed separately and for call rates >90%. Final analyses were performed on 867 cattle and 40 bison with 698,384 SNPs.

### 2.2. Runs of Homozygosity

Following the approach in [9], ROH were estimated using PLINK v1.07 [15] and were defined within a sliding window of 50 SNPs, in one SNP interval, across the genome. Up to one possible heterozygous genotype was permitted and no more than two SNPs with missing genotypes were allowed per window (see [9]).

ROH were divided in seven length categories (1–5 Mb, 5–10 Mb, 10–15 Mb, 15–20 Mb, 20–25 MB, 25–30 Mb, and >30 Mb). For each ROH length category we summed all ROH per animal and averaged this per cattle breed and for the bison. In order to investigate the potential of our approach, we then focused on two length classes: from 500 Kb till 15 Mb to investigate ancient events and >15 Mb to address recent events. To select target chromosomes for detailed analyses, we created Manhattan plots with SAS 9.4 (SAS Institute Inc., Toronto, Canada) for both length classes and selected the chromosomes accordingly. For the chosen chromosomes, we calculated the percentage of times a SNP appeared in a ROH and plotted these results with SAS.

### 2.3. Analyses of Genomic Regions in the Runs of Homozygosity

As an example for the methodology applied in this study, we selected regions of the different chromosomes that showed one of the following patterns (see Figure 2): (a) a simultaneous increase (or decrease) in the number of SNPs in a ROH across all populations, as this pattern could possibly involve genes fundamental for the two species analysed; (b) few populations showing an opposite pattern compared to the others, as this could comprise genes specific for those populations; (c) different patterns between dairy and meat breeds, as this could possibly concern regions under human-induced directional selection; (d) different patterns between bison and domestic cattle breeds, as this pattern may be related to traits important for survival in the wild; (e) a single domestic breed differentiating from the others, as this could relate to specific characteristics of that breed; (f) a long region with a high percentage of ROH, as this could be associated with recent selective events; (g) a short region with opposite trend within a longer homogeneous region, to investigate what could have caused such an abrupt change in variability levels. Each region was screened using NCBI (https://www.ncbi.nlm.nih.gov/) resources for the presence of annotated genes (release 104) and information on their biological function and possible evolutionary importance.

## 3. Results

### 3.1. Runs of Homozygosity

**Figure 1:**
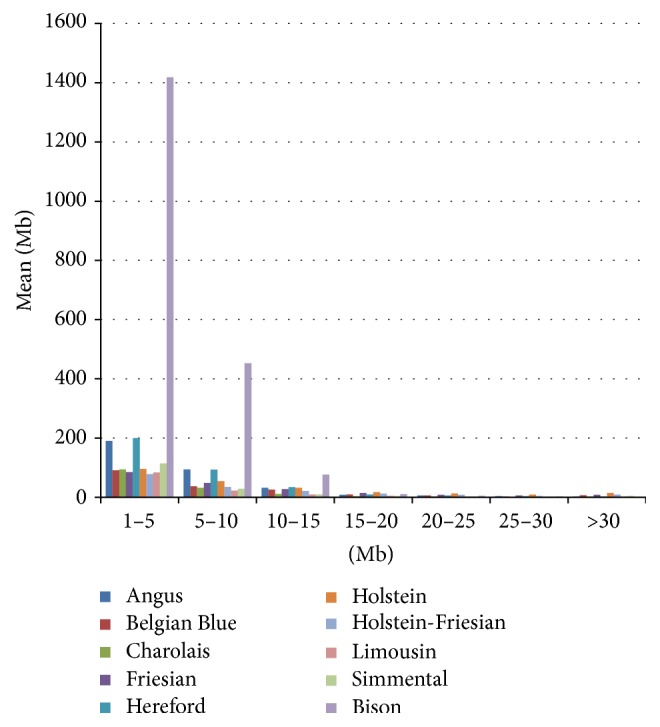
The mean sum of runs of homozygosity (ROH) per genotyped individual, measured in Megabases (Mb) within each population, for each considered ROH length category.

The European bison exhibited the highest mean sum of ROH in the length categories 1–5 Mb, 5-10 Mb, and 10–15 Mb compared to all the domestic breeds. Angus and Hereford also showed considerably higher mean sums than other breeds in the categories 1–5 Mb and 5–10 Mb (see Figure 1).

In the Manhattan plot for the length class between 500 Kb and 15 Mb, chromosomes 2 and 3 showed a group of extremely variable SNPs, while chromosomes 7, 14, and 16 had the highest density and frequencies of SNPs falling in a ROH (see Figure S1a in Supplementary Material available online at http://dx.doi.org/10.1155/2016/2152847). We thus focused on these chromosomes for subsequent analyses. For the ROH >15 Mb, the Manhattan plot showed a more homogeneous distribution but we selected chromosomes 6, 9, and 20 for subsequent analyses (Figure S2a). In the plots based on ROH < 15 Mb, we observed large regions of the bison genome where almost 100% of SNPs fell within a ROH (Figure S1b–f). The frequency of SNPs falling in a ROH > 15 Mb was lower for all populations, in accordance with the smaller number of ROH in this length category (Figure S2b–d). Additionally, the frequency of a SNP falling within a ROH in the bison was not higher than that observed in the domestic breeds with a single exception on chromosome 9 (Figure S2c). On chromosome 20 the highest percentage of SNPs falling within a ROH was detected in dairy cattle breeds (Figure S2d). No clear pattern was observed on chromosome 6 (Figure S2b).

### 3.2. Analyses of Genomic Regions in the Runs of Homozygosity

The in-depth analysis of 17 regions, selected from seven chromosomes (i.e., 2, 3, 7, 9, 14, 16, and 20) led to the identification of more than 300 annotated genes whose functions vary considerably (see Table S1). The most frequent functionally characterised genes were those related to metabolic pathways, but we also observed genes related to disease and immune function, growth, and reproduction. As an example, we review here a few of our observations in the selected regions.

**Figure 2:**
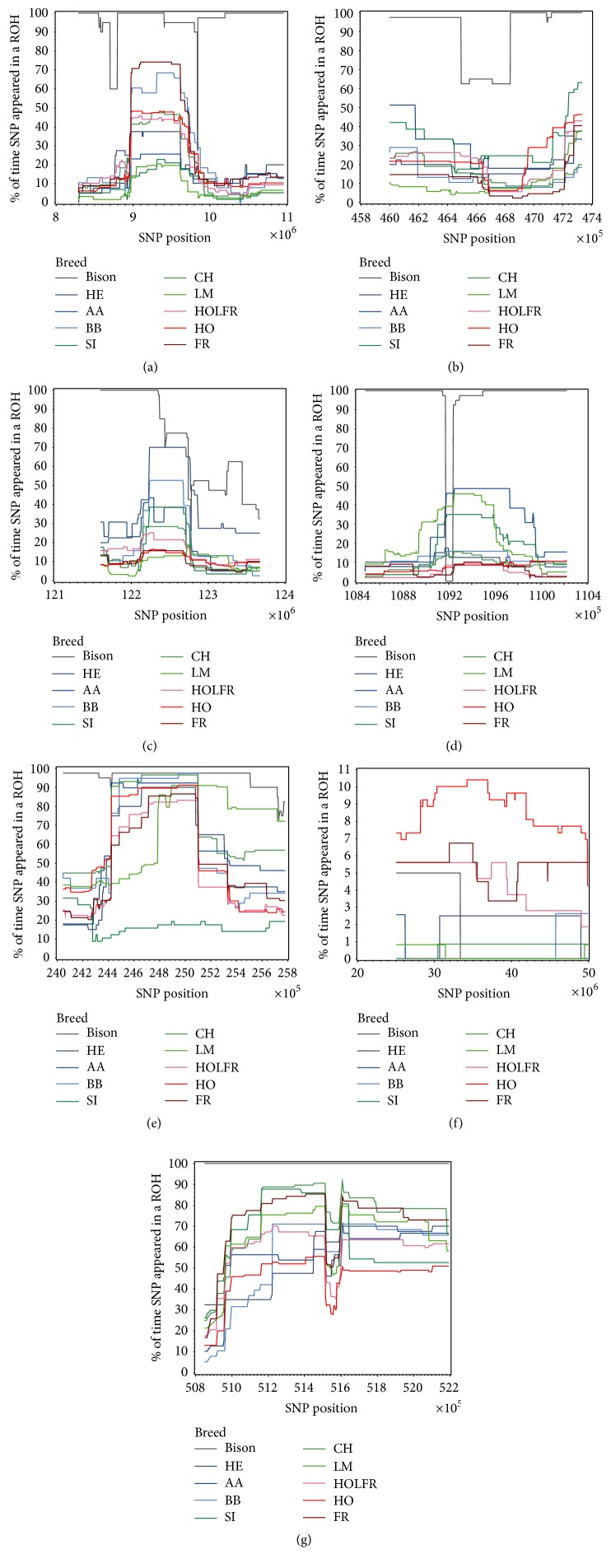
Examples of the investigated patterns. (a) A simultaneous increase (or decrease) in the number of SNPs in a ROH across all populations, as this pattern could possibly involve genes fundamental for the two species analysed (chromosome 3); (b) few populations showing an opposite pattern compared to the others, as this could comprise genes specific for those populations (chromosome 7); (c) different patterns between dairy and meat breeds, as this could possibly concern regions under human-induced directional selection (chromosome 2); (d) different patterns between bison and domestic cattle breeds, as this pattern may be related to traits important for survival in the wild (chromosome 3); (e) a single domestic breed differentiating from the others, as this could relate to specific characteristics of that breed (chromosome 14); (f) a long region with a high percentage of ROH, as this could be associated with recent selective events (chromosome 20); (g) a short region with opposite trend within a longer homogeneous region, to investigate what could have caused such an abrupt change in variability levels (chromosome 7).

In summary, pattern (a) were mainly related to metabolic pathways, involving several CD-, ATP-, and SLAM-family genes (see Table S1) and olfactory receptors. Metabolic pathways were the main genes observed in pattern (b). Pattern (c) was inconclusive for ROH < 15 Mb. In pattern (f) (also an example of (c)) ROH > 15 Mb included genes related to milk and meat quality, growth, and metabolic disorders related to energy unbalanced consumption. Patterns (d) were located in portions of the chromosomes poorly described, with the only exception being the long region on chromosome 9, where a high number of ROH > 15 Mb was observed (Figure S2c). In addition to the metabolism and disease related genes widely encountered in all the screened regions, we report the presence of genes related to olfactory perception, obesity, growth, and sperm malformation in this region. In pattern (e), we observed a region (Figure 2(e)) where the Simmental showed higher variability than the other breeds. Here, genes involved were related to fat thickness and colour, growth, and sperm functionality. In pattern (f), where Hereford showed extremely high frequency values of SNP falling within a ROH and the Belgian Blue extreme variability (with the other breeds in between; Figure S1f, near 45000000), the genes observed were mainly related to the codification of proteins involved in sugar transport and assimilation at cellular level. In pattern (g) we observed genes involved in cortisol pathways and sweet perception, regulation of host response to virus infection, and regulatory function in ovulation.

## 4. Discussion

Our findings revealed several chromosomes with a high number of ROH, and most results concerned ROH < 15 Mb. Upon closer inspection of selected chromosomes, we observed genes potentially important for natural and human-induced selection, concerning, for example, meat and milk quality, metabolism, growth, and immune function. Hence, the ROH approach appears informative for evaluating and comparing species and population history and evaluating possible patterns of adaptation.

We observed comparatively few results for ROH > 15 Mb, the longer regions that are likely to reflect recent inbreeding [9, 11]. Our results may thus suggest relatively limited recent inbreeding in the cattle breeds included in the study, although the many shorter ROH could indicate a lower *N*_E_ in the past [16]. For the European bison, however, large regions of the genome had a 100% (or near 100%) frequency of SNPs falling within a ROH. This suggests high levels of inbreeding, which is consistent with earlier studies and known population history involving a severe bottleneck [17, 18]. However, even limited inbreeding can cause detrimental effects [1, 19] and should be monitored. Earlier studies across species have suggested that ROH > 16 Mb may be considered as recent inbreeding [11, 16]. Analyses of cattle breeds report ROH > 16 Mb as the expected mean after approximately three generations since the most recent common ancestor, whereas autozygosity due to more distant common ancestors will not be captured by this measure [11]. For an in-depth assessment of inbreeding, it may be necessary to investigate different ROH length classes considering the history of the organisms under study. For example, comparisons between wild and domestic species may show different patterns than native and commercial livestock in terms of recent and/or past histories of inbreeding. Consequently, ROH length classes should be assessed on a case by case basis with exploratory analyses informed, where possible, by the history of the species under study.

Variation in sample size and *N*_E_ may have influenced the results. Our comparison of, for example, Belgian Blue (*n* = 38) and Holstein (*n* = 262) should therefore be interpreted with caution. Other important factors that may play a role are differences in breed genetic diversity. McTavish et al. [20] reported observed heterozygosity for several breeds included in our study based on 50 K SNP markers. Among the breeds that showed distinct ROH patterns in our study, they note that Simmental showed a heterozygosity of 0.28 (*n* = 77), the Belgian Blue 0.30 (*n* = 4), the Hereford 0.29 (*n* = 98), and the Holstein 0.30 (*n* = 85). Furthermore, the value for Limousin was 0.29 (*n* = 100) and for Charolais was 0.31 (*n* = 53). Although these values are similar despite variable sample size, among- and within-breed variation in genetic diversity could affect ROH results and their interpretation and may therefore complicate our comparison of cattle breeds and European bison.

Angus and Hereford breeds, together with bison, show high mean sum of ROH in the length class 1–10 Mb, which may be a result of ancestral relatedness owing to small founder populations and isolated origins [11]. In particular, the ROH for the bison is extremely high for the intervals 1–5 Mb and 5–10 Mb with several regions that are completely fixed. This appears consistent with an estimated *N*_E_ of 23 and a total of seven founders for the European bison's Lowland population [18]. In comparison, a recent survey presented considerably larger but variable census population size (*N*_C_) and *N*_E_ for some of the cattle breeds included in our study [21]. For Aberdeen Angus, they reported *N*_C_ > 10 M and *N*_E_ of 136. For Holstein, *N*_C_ was >65 M and *N*_E_ was 99, whereas for Limousin, *N*_C_ was >4 M and *N*_E_ was 174. There may thus be considerable differences in population history among breeds and also for breeds within the same group (such as meat production), which could have affected our results.

We observed genes grouped into various functional categories. The types of genes observed may reveal adaptive patterns and indicate human-induced and/or natural selection, for example, in cases of genes linked to growth and immunity where the first is likely to be human-modified and the second is subject to stronger natural selection. Our results also highlight the need to consider potential conflicts between these two sources of selection. For example, we noted a gene implicated in ketosis (region F, chromosome 20), a metabolic disorder that occurs in cattle when energy demands such as high milk production exceed energy intake and result in a negative energy balance. Strong directional selection for high-performance characteristics such as high milk yield may therefore have implications for animal health and welfare, life expectancy, and the ethical dimensions of animal breeding to cope with their living environments (see, e.g., [22, 23]).

### 4.1. Applications

The ROH approach seems informative for investigating selection and evolutionary histories across a range of different populations, including wild/domestic species, native/commercial livestock, and commercial breeds of various kinds (e.g., cattle breeds for milk or meat, sheep breeds for meat or wool). Our study compared cattle with one related wild species, the European bison. However, this species is highly inbred and has low genetic diversity [18]. Study of other wild-domestic species pairs may therefore provide a more nuanced picture of genomic regions under selection, for example, in domestic pigs and wild boar, or captive and free-living populations of the wild boar (e.g., [24]), thus taking advantage of recent developments in high-density genomic arrays to investigate domestic and wild species (e.g., [25]).

The results of our analyses may also suggest applications for genetic rescue. This could include key genetic regions of high variability observed in one breed, which could be transferred to one or more other populations, for example, related to immune system function or tolerance to environmental factors such as heat, parasites, and infectious disease [26, 27]. Moreover, genes related to growth may have important applications for animal breeding and could be introduced to new breeds to enhance both genetic variation and production [28]. Further research may also help clarify the extent to which selection for rapid growth might conflict with selection for meat quality, which may be relevant to conservation management and breeding for both commercial and native livestock breeds (e.g., [29]).

It will be important to establish whether ROH are under selection. If a ROH is not under selection, its length should normally decrease with every generation as the expected length of autozygous segments identical by descent follows an exponential distribution with mean equal to 0.5*g* Morgans, where *g* is the number of generations since the common ancestor [30]. Conversely, a ROH could contain recessive variants that are expressed in the autozygous state. These variants are known to cause various genetic diseases in humans as a result of specific mutations (e.g., phenylketonuria, Tay-Sachs disease, and cystic fibrosis) and may also be involved in complex diseases such as heart and liver diseases and diabetes [31].

For livestock, the incidence of disease associated with intensive production has increased among several breeds [32], such as Holstein and Jersey [33–35]. Additionally, important traits, such as adaptation to low-quality food resources, parasites, and tolerance to disease and temperature fluctuations may be found mostly in native breeds [36]. An important aspect of the ROH assessment will be identification of genetic variants with applications for genetic rescue, which could benefit both native and commercial breeds [28] to increase robustness and tolerance to environmental variation [27, 36].

### 4.2. Possible Limiting Factors

Ascertainment bias could have affected the comparison of ROH between different species (here cattle and bison) [37]. Moreover, our observations are necessarily incomplete, as there are still large regions of the genome that have not been fully described, as testified by the high number of uncharacterised genes we encountered in our screening (see Table S1). However, key genomic regions can be noted for further research, which also helps identify high-priority areas of the genome for future study.

## 5. Conclusions

The comparative methodology presented here permits visual identification of regions of interest, which could be of value for selection and breeding programs. The ROH approach offers several immediate applications. Firstly, breeding strategies may be improved by reduction in ROH that are acting to reduce genomic diversity. Such a strategy could be useful where genomic regions have lost important diversity or been accidentally fixed, for example, as a consequence of a population bottleneck and/or founder effect. Further, the ROH approach has implications for genetic rescue and the design of breeding strategies for populations at risk. The presence of ROH at intermediate frequency in a population may indicate heterogeneity of the *N*_E_ in different genomic regions. Accordingly, a breeding strategy based on maximising *N*_E_ for a population could produce an increase of *N*_E_ for some chromosomal regions and a reduction in others. This situation could complicate the design of a long-term protocol because of the risk of fixation of certain genes and loss of genetic diversity. Human-driven breeding could also overwhelm natural selective pressures, especially for populations mainly governed by genetic drift due to the small *N*_E_. It is therefore necessary to balance various considerations for long-term conservation breeding, and information from ROH can help pinpoint important genomic regions even if we do not, at the moment, have a complete understanding of their function.

## Supplementary Material

FIGURE S1: Manhattan plot and plots of frequency of SNP in a ROH in the range 500Kb - 15Mb. Chromosomes 2, 3, 7, 14 and 16 are shown. FIGURE S2: Manhattan plot and plots of frequency of SNP in a ROH in the range > 15Mb. Chromosomes 6, 9 and 20 are shown. TABLE S1: Summary table showing the genes identified in the screened regions, their function and NCBI description.
