# Supplementary material for: Novel Graphical Analyses of Runs of Homozygosity among Species and Livestock Breeds
Source: Int J Genomics. 2016 Oct 30;2016:2152847. doi: 10.1155/2016/2152847 (PMC5107238; doi:10.1155/2016/2152847)

Figure S1: ROH in the range 500Kb - 15Mb. a) Manhattan plot. Frequency of SNP in a ROH on chromosome b) 2, c) 3, d) 7, e) 14 and f) 16.

a)


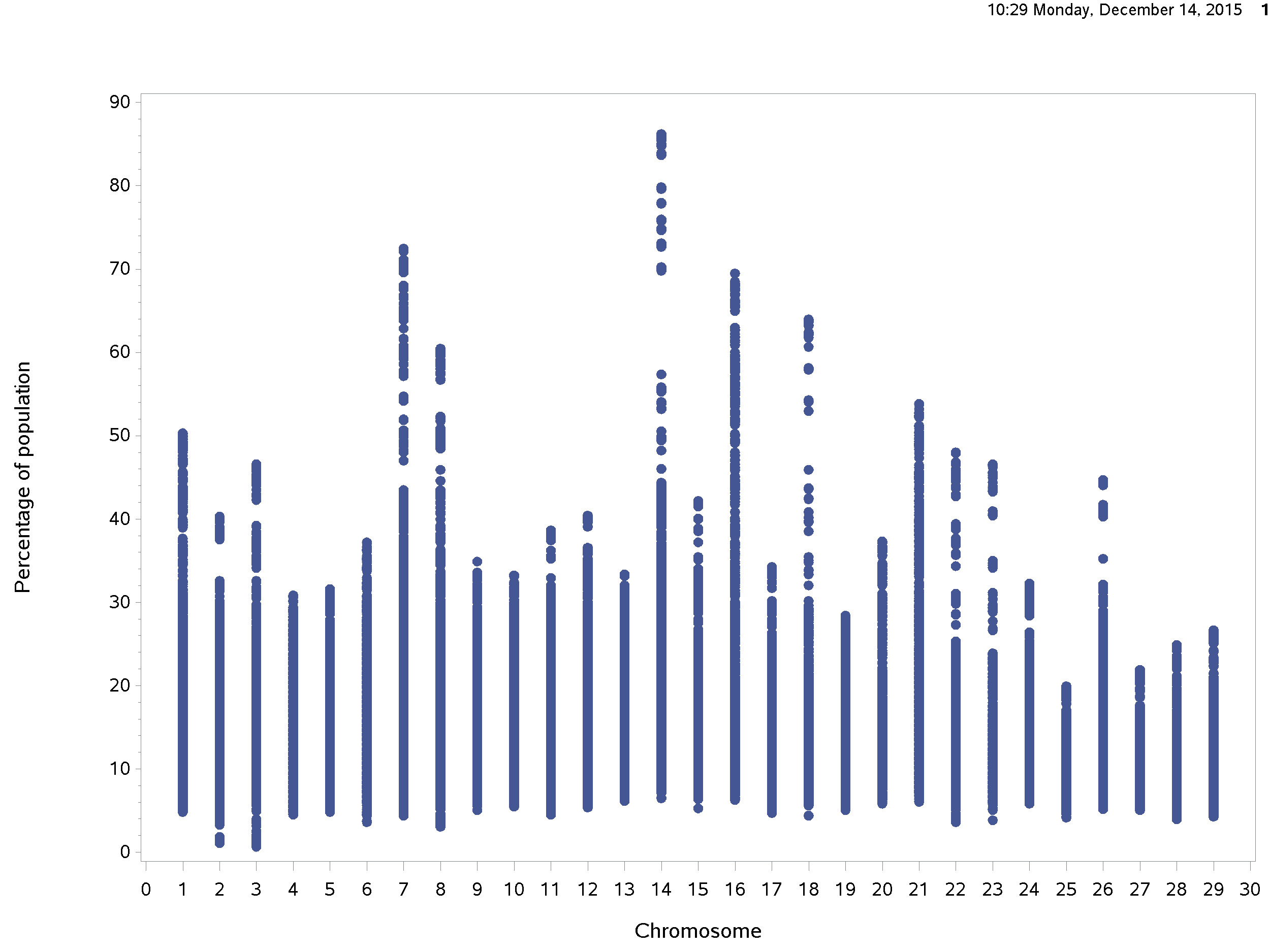


b)


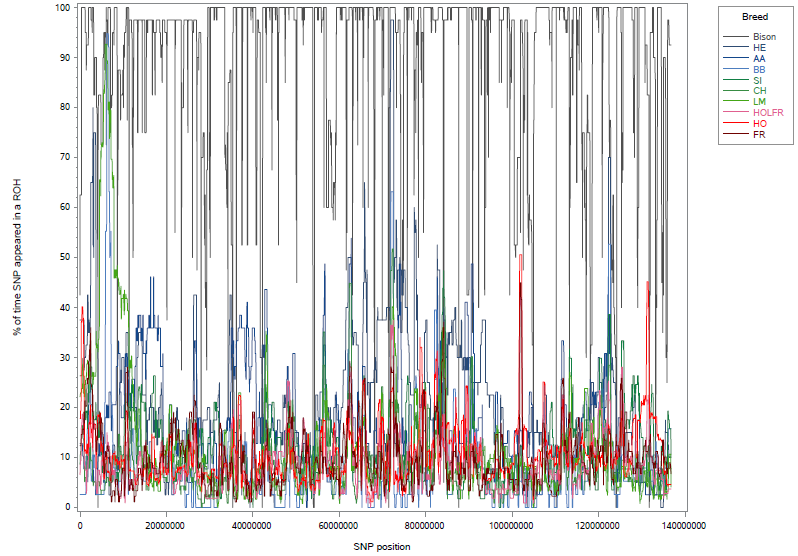


c)
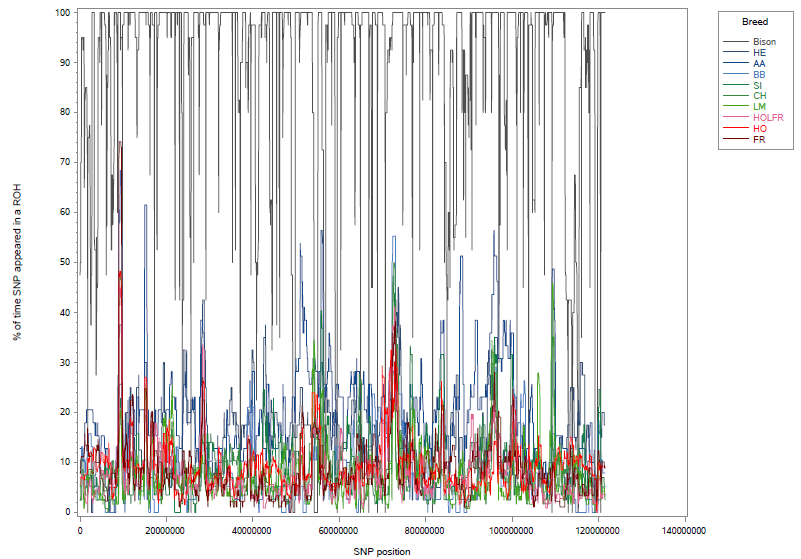


d)
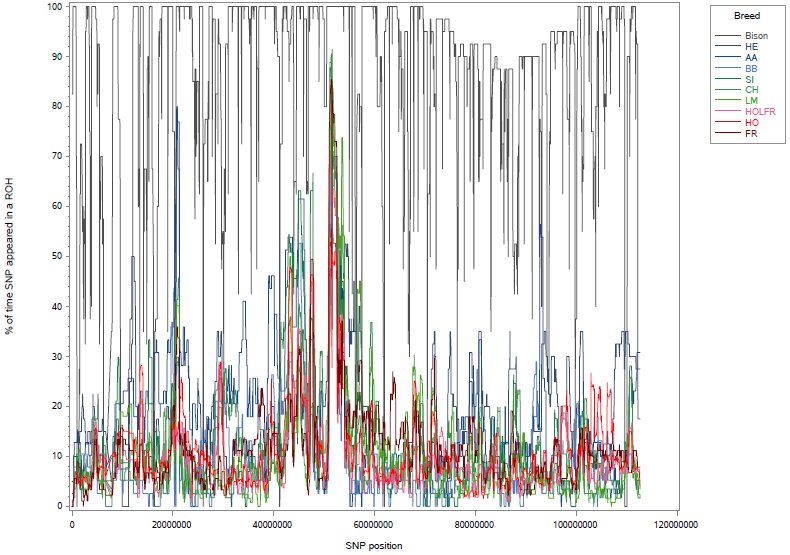


e)
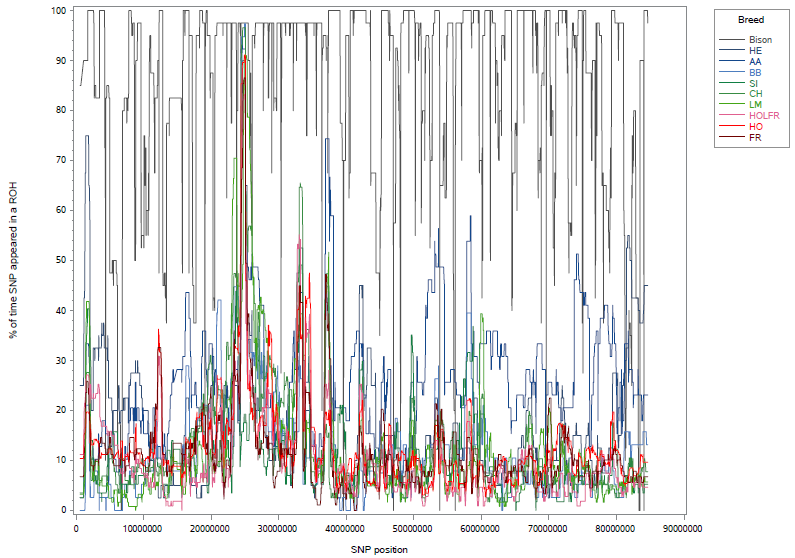


f)
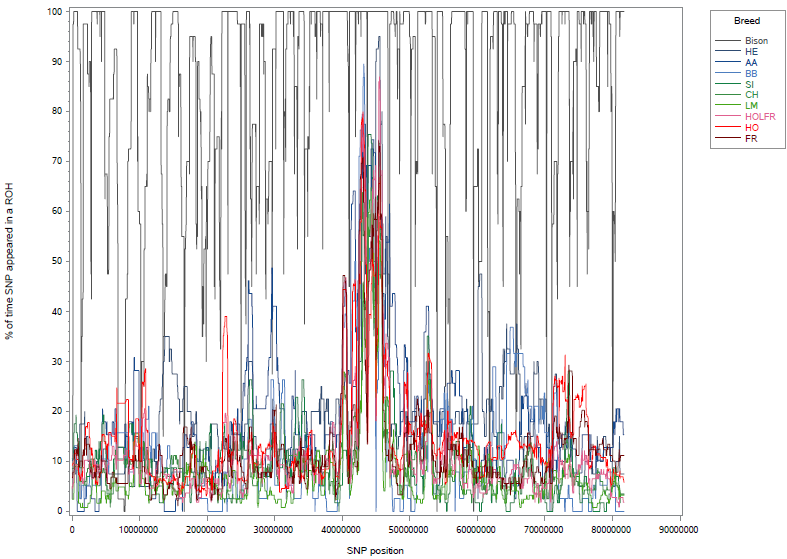


Figure S2: ROH in the range > 15Mb. a) Manhattan plot. Frequency of SNP in a ROH on chromosome b) 6, c) 9 and d) 20.

a)


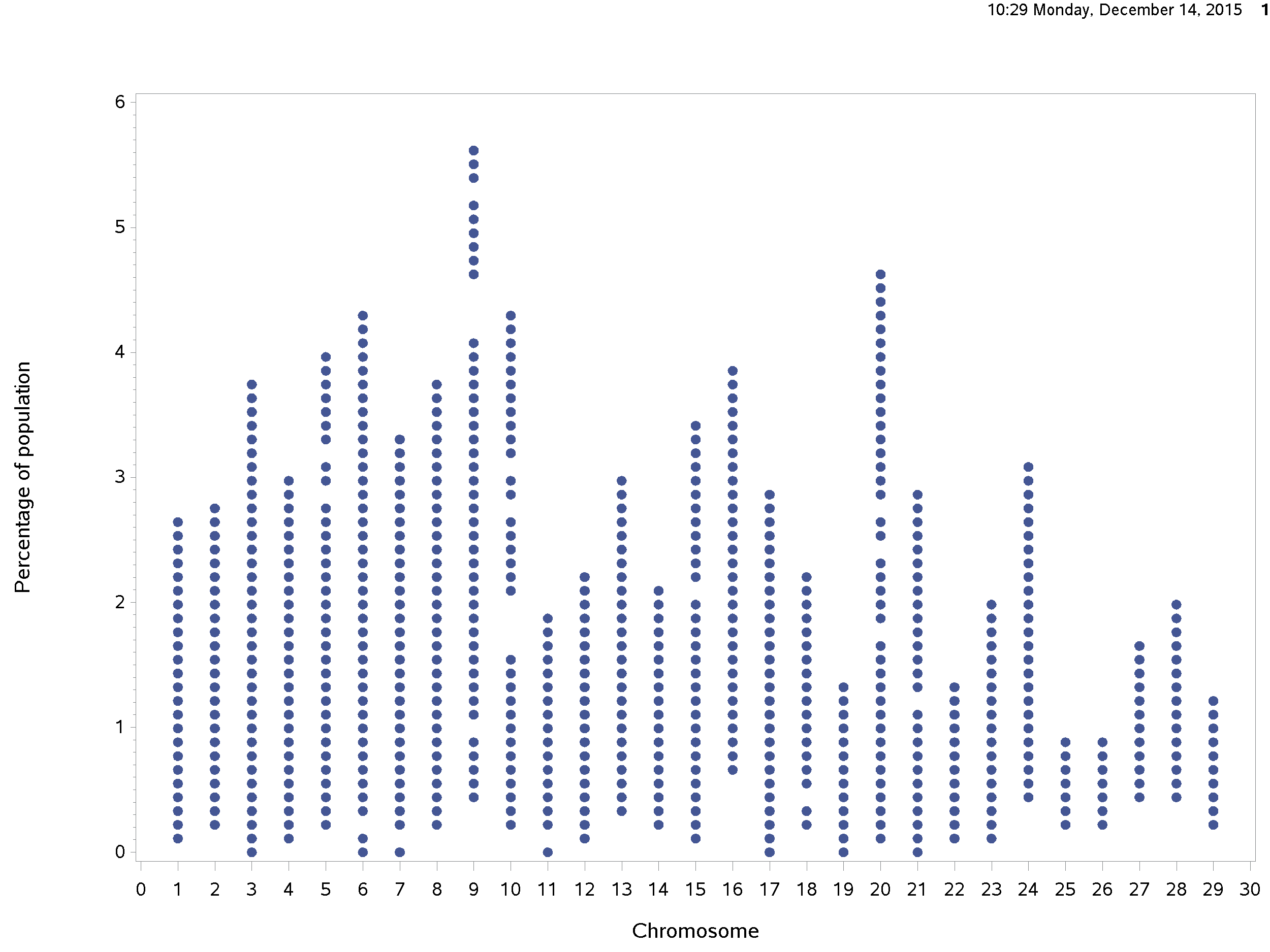


b)
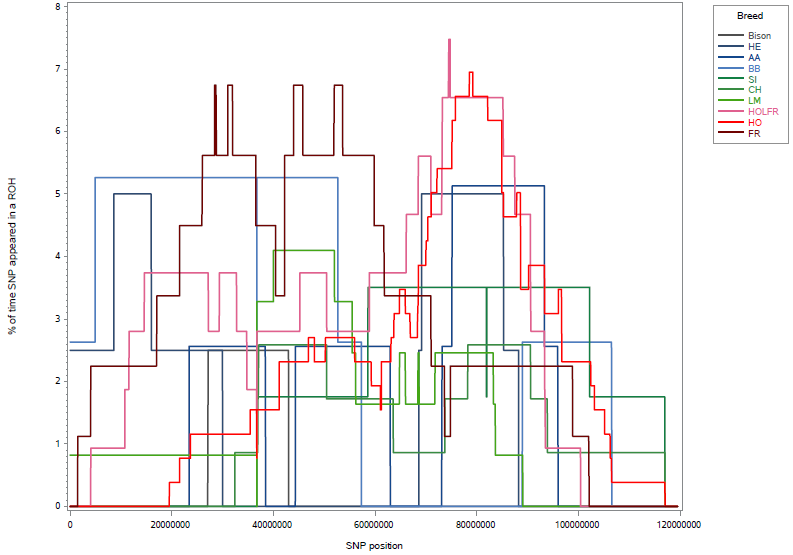


c)
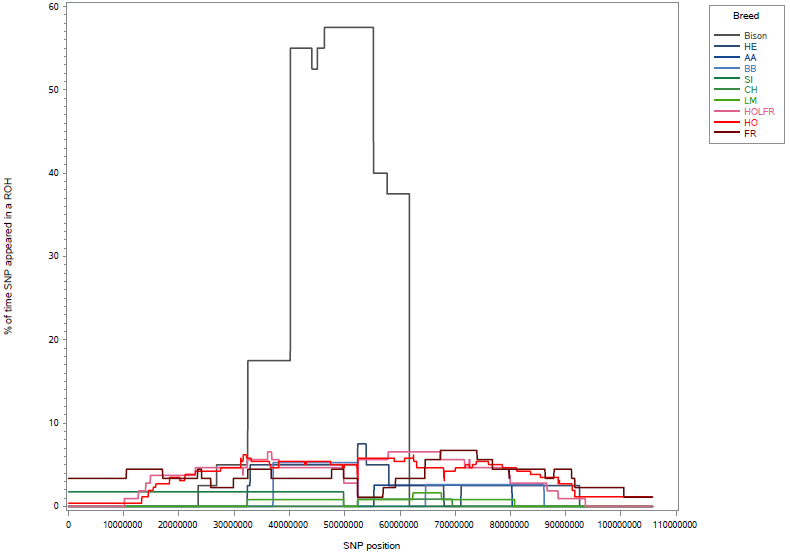


d)
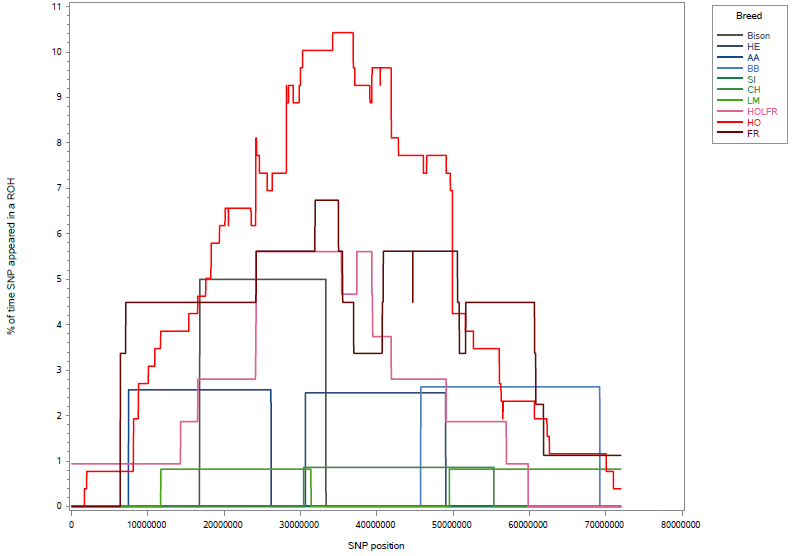

Supplement: Supplementary file 1 — FIGURE S1: Manhattan plot and plots of frequency of SNP in a ROH in the range 500Kb - 15Mb. Chromosomes 2, 3, 7, 14 and 16 are shown. FIGURE S2: Manhattan plot and plots of frequency of SNP in a ROH in the range > 15Mb. Chromosomes 6, 9 and 20 are shown. TABLE S1: Summary table showing the genes identified in the screened regions, their function and NCBI description. [file 2152847.f1.docx]
